# Early Clinical Manifestations Associated with Death from Visceral Leishmaniasis

**DOI:** 10.1371/journal.pntd.0001511

**Published:** 2012-02-07

**Authors:** Valdelaine Etelvina Miranda de Araújo, Maria Helena Franco Morais, Ilka Afonso Reis, Ana Rabello, Mariângela Carneiro

**Affiliations:** 1 Laboratório de Epidemiologia de Doenças Infecciosas e Parasitárias, Pós-graduação em Parasitologia, Departamento de Parasitologia, Instituto de Ciências Biológicas, Universidade Federal de Minas Gerais, Belo Horizonte, Minas Gerais, Brazil; 2 Secretaria Municipal de Saúde, Prefeitura de Belo Horizonte, Belo Horizonte, Minas Gerais, Brazil; 3 Departamento de Estatística, Instituto de Ciências Exatas, Belo Horizonte, Minas Gerais, Brazil; 4 Laboratório de Pesquisas Clínicas, Centro de Pesquisas René Rachou, Fundação Oswaldo Cruz, Belo Horizonte, Minas Gerais, Brazil; 5 Pós-graduação em Ciências da Saúde, Infectologia e Medicina Tropical, Faculdade de Medicina, Universidade Federal de Minas Gerais, Belo Horizonte, Minas Gerais, Brazil; George Washington University, United States of America

## Abstract

**Background:**

In Brazil, lethality from visceral leishmaniasis (VL) is high and few studies have addressed prognostic factors. This historical cohort study was designed to investigate the prognostic factors for death from VL in Belo Horizonte (Brazil).

**Methodology:**

The analysis was based on data of the Reportable Disease Information System-SINAN (Brazilian Ministry of Health) relating to the clinical manifestations of the disease. During the study period (2002–2009), the SINAN changed platform from a Windows to a Net-version that differed with respect to some of the parameters collected. Multivariate logistic regression models were performed to identify variables associated with death from VL, and these were included in prognostic score.

**Principal Findings:**

Model 1 (period 2002–2009; 111 deaths from VL and 777 cured patients) included the variables present in both SINAN versions, whereas Model 2 (period 2007–2009; 49 deaths from VL and 327 cured patients) included variables common to both SINAN versions plus the additional variables included in the Net version. In Model 1, the variables significantly associated with a greater risk of death from VL were weakness (OR 2.9; 95%CI 1.3–6.4), *Leishmania*-HIV co-infection (OR 2.4; 95%CI 1.2–4.8) and age ≥60 years (OR 2.5; 95%CI 1.5–4.3). In Model 2, the variables were bleeding (OR 3.5; 95%CI 1.2–10.3), other associated infections (OR 3.2; 95%CI 1.3–7.8), jaundice (OR 10.1; 95%CI 3.7–27.2) and age ≥60 years (OR 3.1; 95%CI 1.4–7.1). The prognosis score was developed using the variables associated with death from VL of the latest version of the SINAN (Model 2). The predictive performance of which was evaluated by sensitivity (71.4%), specificity (73.7%), positive and negative predictive values (28.9% and 94.5%) and area under the receiver operating characteristic curve (75.6%).

**Conclusions:**

Knowledge regarding the factors associated with death from VL may improve clinical management of patients and contribute to lower mortality.

## Author Summary

The visceral leishmaniasis (VL) is a disease potentially fatal if not diagnosed and treated opportunely. This article presents the results of the study on the manifestations identified at the time of the clinical suspicion of the VL cases. This study was conducted in Belo Horizonte, the capital of the State of Minas Gerais, located in southeastern Brazil. This study is both timely and substantive because the Belo Horizonte is an area of transmission of VL, with one of the highest VL-death proportions of Brazil. The patients with higher risk of death had at least one of the following characteristics: ≥60 years, weakness, HIV co-infection, bleeding, jaundice and other associated infections. During the period 2002–2009, 8% to 22% of the patients with VL progressed to death in Belo Horizonte, whilst the proportion in the country was much lower and varied between 5% and 9%. This study has identified vulnerable patients who are at higher risk of death from VL and who would benefit from early predictive evaluation of the prognostic. Hence, the knowledge regarding the factors associated with death may contribute for clinical management and for reduction of deaths from VL.

## Introduction

The number of new cases of visceral leishmaniasis (VL) is estimated to be around 500,000 per year worldwide with over 50,000 deaths [1]. The majority (>90%) of cases is concentrated in six countries, namely, Bangladesh, Brazil, Ethiopia, India, Nepal and Sudan [2]. In Latin America, the causative agent of VL is the intracellular protozoan *Leishmania infantum* (syn. *L. chagasi*) [3], and the disease is systemic and characterized clinically by prolonged fever, weight loss, hepatomegaly, splenomegaly, hypergammaglobulinemia and pancytopenia. In the absence of treatment, the disease may have fatal consequences [4]. Additionally, the susceptibility to VL, and consequently the epidemiology of the disease, has been influenced by the expansion of human immunodeficiency virus (HIV) in South America, Asia and Africa. Indeed, of the 88 countries that are endemic for VL, 35 have already reported cases of *Leishmania*-HIV co-infection [5]. On this basis, VL is considered an extremely serious public health problem [1], [2].

In Brazil since the 1980's the geographical distribution of VL has expanded, partly due to increased urbanization [1], [2], [6]–[14]. In the 1980s, an average of 1,500 cases was reported each year in Brazil and between 2000–2009 the average increased to 3,480 cases annually, an increase of 132% [13].

In fact, between 1990 and 2009, a total of 57,973 clinical cases of VL had been reported [13], representing 90% of all cases notified in the American continent. These figures do not take into account the unreported cases, which are not insignificant [1], [3], [6], [15], [16].

According to the Visceral Leishmaniais Control and Surveillance Program (VLCP) of Brazil [17] all suspected and confirmed cases of VL must be notified to the sanitary authorities and registered in the Reportable Disease Information System (SINAN, Brazilian Ministry of Health). This system not only provides a center for the collection and processing of data, but also for the dissemination of information generated by the epidemiological surveillance systems linked to the municipal, state and federal governments. Moreover, the SINAN contributes to the knowledge of morbimortality worldwide by VL, since it helps make up the consolidated data from institutions such as PAHO (Pan American Health Organization) and WHO (World Health Organization).

The control of VL in urban areas of Brazil represents, however, a difficult and continuous challenge despite the measures adopted by the Brazilian Ministry of Health, and implemented through of the VLCP [17], which emphasizes the early diagnosis and treatment of clinical cases [18]. In most countries, pentavalent antimonial drugs has been the first choice treatment for more than 70 years [2]. Although more specific guidelines for the management of patients suffering from severe VL have been developed in Brazil [19], the case fatality rate remains high [14]. In Belo Horizonte, the capital of the State of Minas Gerais, the VLCP guidelines have been followed since 1993 but death from VL has not been reduced. In fact, during the period 2002–2009 the case fatality rate of VL in Belo Horizonte ranged from 8.2% (in 2007) to 22.0% (in 2009) with an average of 12.6%, whilst the rate in the country was much lower and varied between 5.6% (in 2008) and 8.5% (in 2003) with an average of 7.0% [14].

Since reduction of case fatality rate is one of the goals of VLCP [17], it is important to understand the determinants of such a poor result in a metropolis presenting active transmission of *L. infantum* [20]. Hence, the objective of the present study was to investigate the early clinical manifestations associated with death from VL using information available from the SINAN database obtained at the moment of clinical case suspicion. Also a prognostic clinical score was proposed to identify patients at a higher risk of death.

## Methods

### Ethical statement

The study was approved by the Ethical Review Board of the Universidade Federal de Minas Gerais (No.211/09) and of the Municipality Health Service of Belo Horizonte (No.075.2008). Data were analyzed anonymously.

### Study design and population

The study was carried out in Belo Horizonte, the capital of the State of Minas Gerais, located in southeastern Brazil, an area comprising 2,375,444 inhabitants [21]. This historical cohort study was based on secondary VL data from 2002 to 2009. Owing to the prolonged incubation period of the disease, data for 2009 were only finalized in March 2010 and, hence, the complete set of information for that year was not available at the time of the study. Data were obtained from the Reportable Disease Information System-SINAN (Brazilian Ministry of Health and Municipality Health Service of Belo Horizonte) and complemented with data of the Mortality Information System-SIM (Brazilian Ministry of Health and Municipality Health Service of Belo Horizonte). The selection criteria for inclusion in the study were: (i) the patient was resident in Belo Horizonte; (ii) the patient represented a new case of VL; (iii) the case was registered at SINAN, and, if appropriate, (iv) the primary cause of death of the patient was VL. Based on these criteria, 888 VL patients (92% of all of those registered) were selected for the study and, of these, 111 died (88% of all deaths from VL.

### SINAN database

The epidemiological surveillance system of the Brazil involved registration of the suspected VL case at SINAN using a form comprising the following entries: date of notification, health unit responsible for notification, address, age, sex, level of schooling, occupation of patient, date of the start of symptoms and clinical manifestations (signs and symptoms). Subsequently, further information was added to the records including the results of specific laboratory examinations, date of beginning of treatment, initial drug used for treatment, drug used following failure of the initial therapy, and evolution of the case.

The SINAN database changed platform during the study period from a Windows-based version (2002–2006) to a Net version (2007–2009). As shown in Table 1, the Net version contained more information than the Windows version except for the field relating to co-infections, which was simplified (HIV remained but tuberculosis was removed). Of the 888 cases, 512 had been registered in the Windows version and 376 in the Net version. Data from the two versions of the SINAN database was combined in order to create a single database, and the consistency of the data contained therein evaluated. The variables analyzed in the present study were sex and age of patient, clinical manifestations (signs and symptoms), co-infections (HIV and tuberculosis), date of notification, date of the onset of symptoms, initial drug administered, duration of treatment with pentavalent antimonial drugs, other drug administered following failure of initial therapy, evolution of the case (cure or death from VL) and date of death (if applicable). Those variables exhibiting high levels of missing information, such as schooling (55.5%), ethnicity (51.8%), weight (83.8%), occupation (85.0%), date of the start of treatment (69.1%) and relapse (86%) were excluded from the study.

**Table 1:** Comparison between the variables of Windows and Net versions of the SINAN database.

Field	Version Windows	Version Net
**Clinical manifestations** (Yes, No, Unknow)	Fever, weakness, emaciation, cough and/or diarrhea, splenomegaly, hepatomegaly	Added variables: swelling, pallidness, other infections, bleeding, jaundice and others manifestations
**Co-infections** (Yes, No, Unknow)	HIV, tuberculosis, other types of infection	HIV co-infection only
**Date of the beginning of treatment**	Inexistent field	Existing field
**Diagnosis confirmation criteria** (laboratory, clinical-epidemiological)	Inexistent field	Existing field
**Evolution of the case**	Cure, death, unknown	Cure, abandoned treatment, VL-death, death by other causes, transfer (treatment interrupted in the original municipality, but continued at some other location)

### Statistical analysis

Statistical analyses of the data were performed using STATA version 11.0 software (Stata Corp., College Station, TX, USA) considering 111 deaths from VL and 777 cured patients. Univariate logistic regression analysis was used to evaluate the demographic and clinical variables according to the occurrence of death from VL. Variables associated with death from VL at a significance level of *p*<0.25, along with those previously considered in the literature to be biologically important in the occurrence of death from *L. infantum*, were included in multivariate logistic regression analyses. Variables with more than two categories were transformed into dummies variables.

Two multivariate logistic regression models were subsequently analyzed. Model 1 covered the period 2002 to 2009 and included the variables present in both versions of SINAN (111 deaths and 777 cured patients), whereas Model 2 covered the period 2007 to 2009 and included the variables common to both versions of SINAN together with the new variables included in the Net version (49 deaths and 327 cured patients). With the aim of avoiding selection bias, and to allow better adjustment of the model to VL data, the category “unknown” was maintained for variables for which information was missing. Variables presenting collinearity were evaluated and those that better explained the occurrence of death from VL were retained in the model.

A step-by-step backward selection procedure was used to select the variables and to produce the final multivariate logistic regression models. Only adjusted variables showing a significant association (*p*<0.05) with the occurrence of death from VL remained in the final models. The strength of association was determined by odds ratio at a 95% confidence interval.

The predictive factors relating to death from VL that were identified by Model 2 (period 2007–2009) were used to create a prognosis score. According to the methodology described by Barquet et al. [22], the regression coefficient of each of the variables was divided by the smallest coefficient and the quotient was rounded to the nearest integer in order to facilitate the clinical use of the system. The prognosis score was validated against all cases registered during the period 2007–2009. The actual evolution of each patient, defined as death from VL (yes *versus* no), was compared with the predictive score. For the purposes of comparison, patients attaining a predictive score in the range from 1 to 5 received an allocated score of 1, while those presenting none of the death prognosis factors received an allocated score of 0. The predictive performance of the score was determined by sensitivity, specificity, positive and negative predictive values, and the area under the receiver operating characteristic curve (ROC) [23].

## Results

The incidence of VL in Belo Horizonte during the period 2002–2009 varied from 3.4 to 6.6/100,000 inhabitants and the case fatality rate was 13.1% (Table 2). The highest levels of lethality were observed in 2009 (22.0%) followed by 2004 (18.7%). The ages of the 888 VL subjects varied between 3 months and 93 years; the ages of those that died from VL ranged from 5 months to 86 years (*n* = 111; median = 18 years; interquartile range = 4−41 years), whereas the ages of cured patients varied from 3 months to 93 years (*n* = 777; median = 32 years; interquartile range = 7−54 years). Case fatality rate amongst patients presenting *Leishmania*-HIV co-infection was 17.7% (12/68), while lethality amongst those with *Leishmania*-tuberculosis co-infection was 10.5% (4/38). Of the nine patients presenting both types of co-infection (*Leishmania*-HIV-tuberculosis), four progressed to death (44.4%). The number of deaths from VL and the case fatality rate was higher in patients ≥60 years (24.4%), followed by those within the age range 30–39 years (20.2%) (Table 3).

**Table 2:** Visceral leishmaniasis incidence and case fatality rates, Belo Horizonte, Brazil, 2002–2009.

Year	VL Cases^a^ *(n)*	Incidence^a^^,^ b *(per 100, habitants)*	Deaths	Case fatality rate *(%)*
2002	77	3.4	9	11.7
2003	103	4.5	10	9.7
2004	134	5.8	25	18.7
2005	110	4.6	10	9.1
2006	128	5.3	12	9.4
2007	110	4.5	9	8.2
2008	161	6.6	20	12.4
2009	141	5.8	31	22.0
Total	964	..^c^	126	13.1

a Cases obtained from the Municipality Health Service of Belo Horizonte/SINAN.

b Population data from Brazilian Institute of Geography and Statistics/IBGE.

c Numerical information not applicable.

**Table 3:** Visceral leishmaniasis cases and fatality rate by age and sex, Belo Horizonte, Brazil, 2002–2009.

Age range(years)	Sex								Total			
	Female				Male							
	Cases		Deaths	Case fatality rate (%)	Cases		Deaths	Case fatality rate (%)	Cases		Deaths	Case fatality rate (%)
	n	%			n	%			n	%		
0–4	112	31.9	9	8.0	120	22.4	11	9.2	232	26.1	20	8.6
5–9	56	16.0	3	5.4	54	10.1	7	13.0	110	12.4	10	9.1
10–19	38	10.8	1	2.6	51	9.5	2	3.9	89	10.0	3	3.4
20–29	40	11.4	3	7.5	70	13.0	13	18.6	110	12.4	16	14.6
30–39	23	6.6	4	17.4	71	13.2	15	21.1	94	10.6	19	20.2
40–49	36	10.3	6	16.7	69	12.9	8	11.6	105	11.8	14	13.3
50–59	21	6.0	3	14.3	41	7.6	5	12.2	62	7.0	8	12.9
≥60	25	7.1	5	20.0	61	11.4	16	26.2	86	9.7	21	24.4
Total	351	100.0	34	9.7	537	100.0	77	14.3	888	100.0	111	12.5

The results of univariate logistic regression analysis of the demographic and clinical variables with respect of deaths from VL are shown in Tables 4 and 5. The most significant non-adjusted variables (*p*<0.05) associated with death were weakness, edema, bleeding, other associated infections, jaundice, *Leishmania*-HIV co-infection, *Leishmania*-tuberculosis co-infection, treatment with amphotericin, treatment with pentavalent antimonial drug for 21 to 40 days, male gender, age range 30–39 years and ≥60 years.

**Table 4:** Demographic and clinical variables according to death from visceral leishmaniasis, Belo Horizonte, Brazil, 2002–2009.

Variables (n)^a^	Deaths from VL				Odds Ratio	95% IC	p values
	No		Yes				
	n	%	n	%			
**Sex** (n = 888)							
Female	317	40.8	34	30.6	1.0	..^b^	..
Male	460	59.2	77	69.4	1.6	1.0–2.4	0.04
**Age range** (years; *n* = 888)							
0–4	212	27.3	20	18.0	1.0	..	..
5–9	100	12.9	10	9.0	1.1	0.5–2.4	0.89
10–19	86	11.1	3	2.7	0.4	0.1–1.3	0.12
20–29	94	12.1	16	14.4	1.8	0.9–3.6	0.10
30–39	75	9.7	19	17.1	2.7	1.4–5.3	0.00
40–49	91	11.7	14	12.6	1.6	0.8–3.4	0.19
50–59	54	6.9	8	7.2	1.6	0.7–3.8	0.31
≥60	65	8.4	21	18.9	3.4	1.8–6.7	0.00
**Clinical manifestation**							
Fever (*n* = 860)	723	96.1	102	94.4	0.7	0.3–1.7	0.41
Splenomegaly (*n* = 825)	642	89.0	92	88.5	0.9	0.5–1.8	0.86
Hepatomegaly (n = 827)	607	84.0	88	84.6	1.1	0.6–1.9	0.86
Weakness (*n* = 801)	568	81.3	95	93.1	3.1	1.4–6.9	0.01
Emaciation (*n* = 783)	516	75.0	78	82.1	1.5	0.9–2.7	0.13
Cough (*n* = 742)	319	48.9	50	55.6	1.3	0.8–2.0	0.24
Edema(c) (*n* = 240)	24	11.6	13	39.4	5.0	2.2–11.2	0.00
Pallidness(c) (*n* = 259)	149	66.2	28	82.4	2.4	0.9–6.0	0.07
Other infections^(3)^ (*n* = 236)	44	21.8	19	55.9	4.6	2.1–9.7	0.00
Bleeding(c) (*n* = 238)	16	7.8	11	34.4	6.2	2.6–15.2	0.00
Jaundice(c) (*n* = 238)	13	6.3	14	43.8	11.6	4.7–28.3	0.00
Other manifestations(c) (*n* = 206)	55	31.1	7	24.1	0.7	0.3–1.8	0.45
**Co-infection**							
HIV (*n* = 553)	39	8.0	12	17.7	2.5	1.2–5.0	0.01
Tuberculosis(d) (*n* = 345)	9	2.9	4	10.5	3.9	1.1–13.3	0.03

a The total number of individuals listed in SINAN and included in the study (Belo Horizonte, Brazil) were 888, of which 512 were registered in the Windows version and 376 in the Net version of the database.

b Numerical information not applicable.

c Variable registered for VL cases occurring in the period 2007–2009 (Net version of SINAN).

d Variable registered for VL cases occurring in the period 2002–2006 (Windows version of SINAN).

**Table 5:** Clinical variables according to death from visceral leishmaniasis, Belo Horizonte, Brazil, 2002–2009.

	Deaths from VL				Odds Ratio	95%IC	p values
Variables (n)^a^	No		Yes				
	n	%	n	%			
**Time between the onset of symptoms and notification** (*n* = 888; weeks)							
up 4	441	56.8	54	48.7	1.0	..^b^	..
5–8	144	18.5	21	18.9	1.2	0.7–2.0	0.52
9–12	84	10.8	16	14.4	1.6	0.9–2.9	0.15
>12	108	13.9	20	18.0	1.5	0.9–2.6	0.14
**Treatment**							
**Initial drug administered** (*n* = 753)							
Pentavalent antimonial (*n* = 612)	570	84.8	42	51.9	1.0	..	..
Amphotericin (*n* = 135)	96	14.3	39	48.1	5.5	3.4–9.0	0.00
Pentamidine (*n* = 6)	6	0.9	-c	-	-	-	-
**Duration of treatment with pentavalent antimonial** (*n* = 354; days)							
<20 (n = 40)	32	9.8	8	30.8	1.0	..	..
20 (n = 55)	48	14.6	7	26.9	0.6	0.2–1.8	0.34
21–40 (*n* = 251)	240	73.2	11	42.3	0.2	0.1–0.5	0.00
>40 (*n* = 8)	8	2.4	-	-	-	-	-
**Other drugs used following initial therapy failure** (*n* = 53)							
Pentavalent antimonial (n = 11)	10	21.7	1	14.3	1.0	..	..
Amphotericin (n = 41)	35	76.1	6	85.7	1.7	0.2–16.0	0.64
Pentamidine (n = 1)	1	2.2	-	-	-	-	-

a The total number of individuals listed in SINAN and included in the study were 888, of which 512 were registered in the Windows version and 376 in the Net version of the database.

b Numerical information not applicable.

c symbol equal to zero.

The results of the multivariate logistic regression analysis of those variables that were associated (*p*<0.25) with death from VL, and of those reportedly important for such an outcome, are presented in Table 6. Model 1 (period 2002–2009) showed that weakness, *Leishmania*-HIV co-infection and age ≥60 years were associated with a greater chance of death. Model 2 (period 2007–2009) revealed that variables included in the SINAN Net version, such as other associated infections, bleeding and jaundice, were significantly associated with the increased likelihood of deaths from VL. It is important to emphasize that inclusion of the category unknown in the multivariate logistic regression analysis had no effect on the results generated by models 1 and 2, since the odds ratio and the 95% CI values remained unchanged in either the presence or absence of this category. The use of the category unknown not only allowed a larger number of cases to be considered in the final models but also improved the adjustment of these models to the VL data as shown by the log likelihood and *p* values.

**Table 6:** Factors associated with death from visceral leishmaniasis, Belo Horizonte, Brazil, 2002–2009.

Variables	Odds Ratio (95% CI)	Adjusted Odds Ratio (95% CI)	p values
**Model 1 (** ***n*** ** = 888)** a			
Weakness	3.1 (1.4–6.9)	2.9 (1.3–6.4)	0.01
*Leishmania*-HIV co-infection	2.5 (1.2–5.0)	2.4 (1.2–4.8)	0.02
Age (≥60 *versus*<60 anos)	2.6 (1.5–4.4)	2.5 (1.5–4.3)	0.00
**Model 2 (** ***n*** ** = 376)** b			
Other infections	4.6 (2.1–9.7)	3.2 (1.3–7.8)	0,01
Bleeding	6.2 (2.6–15.2)	3.5 (1.2–10.3)	0.02
Jaudice	11.6 (4.7–28.3)	10.1 (3.7–27.2)	0.00
Age (≥60 *versus*<60 anos)	2.6 (1.5–4.4)	3.1 (1.4–7.1)	0.02

a Covering the period 2002–2009 and including the variables present in both versions of SINAN (111 death from VL and 777 cures).

b Covering the period 2007–2009 and including the variables common to both versions of SINAN together with the new variables included in the Net version (49 death from VL and 327 cures).

Table 7 presents the predictive score, prepared from VL cases notified between 2007 and 2009, for each of the four death-associated factors revealed by Model 2. According to this system, a score of 1 was attributed to the three variables (age ≥60 years, bleeding and other associated infections), while a score of 2 was attributed to the variable jaundice. The performance measures of the predictive score were sensitivity (71.4%), specificity (73.7%), positive and negative predictive values (28.9% and 94.5%) and the area under the ROC curve (75.6%).

**Table 7:** Predictive scoring system for death from visceral leishmaniasis, Belo Horizonte, Brazil, 2007–2009.

Prognosis factor	Regression Coeficient	Score
Other infections	1.17	1
Bleeding	1.25	1
Jaundice	2.31	2
Age (≥60 versus<60 years)	1.13	1

Scoring system based on the variables listed in the SINAN Net version.

## Discussion

In the present study, the factors associated with death from VL were weakness, *Leishmania*-HIV co-infection, other associated infections, bleeding, jaundice, and age ≥60 years. These findings are in agreement with the Brazilian Ministry of Health [12], [17], [19] in which factors associated with death from VL were considered to be age <6 months or >65 years, jaundice, bleeding and co-morbidities including bacterial infections.

The records analyzed in the present study were obtained from a database containing details of registered VL cases, each of which would normally have been notified on clinical suspicion of the disease. Our results reveal that it is possible to detect the factors associated with death from VL at first clinical suspicion of the disease and, hence, to identify the most vulnerable patients. Timely application of specific and effective measures to the patients would contribute greatly to a reduction in the lethality of the disease. Several authors have suggested that knowledge regarding the laboratory and clinical profiles of patients and their association with death from VL could assist in the clinical management and reduce lethality [4], [12], [17], [19], [24]–[26].

The case fatality rate registered in Belo Horizonte is one of the highest in Brazil. Some hypotheses could explain the variation in the rates as accessibility to the health service, suspicion of VL and delayed diagnosis, treatment opportunity, clinical management of the patient, toxicity of drugs, comorbities and *Leishmania* population in circulation in Belo Horizonte. Considering the case fatality rates in 2004 (18.7%) and 2009 (22.0%), it is possible that the complex combination of these factors could contribute to the unacceptable fatality rates during these years.

Factors associated with lethality from VL have been reported in the literature [2], [4], [24]–[28]. A retrospective cohort study [26] conducted in Recife (north-eastern Brazil) identified risk factors associated with VL death in young patients (<15 years old) as mucosal hemorrhage, jaundice, dyspnoea, bacterial infections, reduced number of neutrophils and platelets. A case-control study [4] conducted in Teresina (also in north-eastern Brazil) described the occurrence of fever for more than 60 days, diarrhea, jaundice and anemia as predictive factors for the death in VL patients. Another case-control study performed in Teresina [25] confirmed that bacterial infections and hemorrhage were the most relevant factors associated with death from VL. However, a study performed in Campo Grande (central west Brazil) identified bacterial infections as the main cause of death among VL patients [24]. According to Seaman et al [27], age ≥45 years, disease duration >5 months, undernutrition and intense anemia were associated with an increased risk of death from VL in Sudan. Following a study also performed in Sudan, Collin et al [28] reported that the prognosis factors for death were age <2 or ≥45 years, disease duration >5 months, undernutrition, anemia, splenomegaly and, particularly, episodes of diarrhea, vomit and bleeding.

The time interval between the onset of symptoms and time of diagnosis was estimated and this interval was not significantly associated with death from VL (Table 5). However, other studies show the necessity to reduce delay in diagnosis and describe the association between time of diagnosis and treatment with the VL case outcome (cure or death). According to Kajaia et al [29], the factors associated with VL relapse were delay in diagnosis for >90 days, haemoglobin level <60 g/L and age <1 year. In Tunisia [30], children were evaluated and seven prognostic factors at the time of hospital admission were identified: visit delayed >56 days, fever lasting >21 days, normal or low temperature, hemorrhagic syndrome, hemoglobin rate <5.5 g/dL, sedimentation rate <25 mm and hypoalbuminemia <30 g/L. In Sudan [28], risk factors for death among adults were age ≥45 years, malnutrition, anemia and duration of illness ≥5 months. Also in Sudan [27], the risk factors in adult patients were duration of illness ≥5 months, age ≥45 years, hemoglobin level <60 g/L, and body mass index <12 kg/m^2^.

The weakness, which is one of the earliest clinical symptoms of VL, was found to be significantly associated with death. The most likely explanation for this finding is that the study was based on clinical manifestations presented by VL-suspect patients at their first medical appointment. However, the early detection of weakness may help to identify those patients presenting a higher likelihood of an unsatisfactory evolution of their disease. A study conducted in central west Brazil involving 55 individuals that had died from VL described the occurrence of hyporexia (65.5%), asthenia (58.1%) and adynamia (29.0%) [24]. It is probable that this set of conditions formed part of the weakness reported by patients during anamnesis, thus confirming the findings of the present study.

A VL-suspected case is defined as a patient presenting fever and splenomegaly who originates from an *Leishmania* transmission area or, if originating from an area in which transmission is absent, where differential diagnosis has been discarded [12], [17]. As expected, there were high frequencies of patients in the study population presenting either fever with splenomegaly (87.3%) or fever with splenomegaly and hepatomegaly (83.0%), and the frequencies were similar in death from VL and in cured patients.

The occurrence of jaundice was registered for 27 patients, 14 (51.9%) of whom died from VL. The presence of jaundice increased the chances of death from VL by a factor of 10 (ORadjusted 10.1; 95%CI 3.7–27.2). The large amplitude of the confidence interval can be explained by the small number of patients presenting jaundice. Following a study carried out in the Brazilian State of Piauí involving 12 deaths from VL and 78 cured individuals, Werneck et al. [4] reported that the death-associated factors were anemia, jaundice, fever for more than 60 days and diarrhea. These authors pointed out that the last three symptoms mentioned were identified at the first clinical examination and that the presence of jaundice increased the chances of death by 10.6-fold (95%CI 1.2–94.8). Furthermore, according to a study conducted in the central west State of Mato Grosso do Sul, jaundice was observed in 20.0% of patients who subsequently died from VL [25]. It may be concluded that identification of jaundice at the first examination or during a later follow is a valuable predictor of VL patients likely to present a negative prognosis.

The occurrence of jaundice may indicate liver damage [25] which, in some cases of VL, has been observed in the form of a massive necrotic destruction of the organ [31], [32]. Moderate alterations of hepatic function, together with thrombocytopenia, may give rise to serious hemorrhagic processes [27]. A study of clinical and laboratory data relating to 55 patients who had died from VL revealed increased levels of albumin and aspartate aminotransferase at the time of admission to hospital, and high levels of creatinine and amylase prior to death [24]. According to Jeronimo et al. [10], increased levels of liver enzymes in untreated patients presenting a profile of jaundice at the time of admission to hospital, may signal the presence of hepatitis by *Leishmania* infection.

In the present study, the ages of cured patients varied from 3 to 93 years old whereas the ages of those who had died from VL were between 5 months and 86 years. The lethality in older individuals (≥60 years) was the highest (24.4%), a value similar to that reported by Oliveira et al. [24] for elderly subjects. In Sudan [27], age ≥45 years old was associated with an increased risk of death from VL. It is expected that older VL patients would present a higher risk of mortality for many reasons, *e.g.* cardiovascular diseases that may coexist with leishmaniasis. Additionally, although *N*-methyl glucamine antimoniate constitutes the first choice treatment for VL, the drug may have side effects including severe cardiac arrhythmia. This medication was prescribed for 50 of the patients aged ≥60 years included in the present study, and seven (14.0%) of these died. However, other studies [25], [28] associated a higher risk of death with those of a younger age. In Uganda [33], the main risk factors for in-hospital death identified were age <6 years and >15 years, concomitant tuberculosis or hepatopathy, and drug-related adverse events. The case fatality rate among patients >45 years of age was strikingly high (29.0%).

Multivariate regression analysis models 1 and 2 identified age of ≥60 years as a factor associated with death from VL (OR 2.5 and 3.1, respectively). It is worth noting that the present study involved 86 patients aged ≥60 years and of these 21 died from VL accounting for 19.0% of the total deaths analyzed. However, in 2009, the Brazilian Ministry of Health mentioned that VL patients aged <1 and >40 years were at greater risk [12], and more recently, Costa et al. [25] described that individuals in these two age ranges are the most vulnerable to death from VL.

In this study, 63 subjects presented other associated infections with significantly difference between the cured patients (n = 44; 5.7%) and those that died from VL (n = 19; 17.1%) Multivariate logistic regression analysis showed that the occurrence of other infections increased the chances of death by 3-fold. A recent study involving 55 hospitalized VL patients who progressed to death found that 65.5% had been diagnosed with other infections, most commonly sepsis (66.7%) and pneumonia (63.9%), at the time of admission and during hospitalization [24]. The most likely explanation for such a high frequency of co-infection is that all of the studied patients were hospitalized and could be evaluated at the time of admission and throughout hospitalization. Patients with VL are characteristically neutropenic and, therefore, present reduced inflammatory response and are at increased risk from other established or concealed infections [34]. This type of physiopathological mechanism may explain the inclusion of other infections among the prognosis factors for death from VL.

Twenty-seven individuals presented history of bleeding at the first clinical examination with frequencies that were significantly different between the 16 cured patients (2.1%) and the 11 deaths from VL (9.9%). The presence of bleeding increased the chances of death by 3.5-fold. Oliveira et al. [24] recently reported the occurrence of bleeding in 32.7% of deaths from VL. Infection and hemorrhage are classical complications of VL [4], [12], [19], [24], [25] and, in the present study, were recorded in 19 and 11 deaths, respectively. Twelve patients were affected by both complications, and of these six (50.0%) died from VL. In a case-control study, Werneck et al. [4] showed that all of the cases of death from VL presented infectious or hemorrhagic complications, while Costa et al. [25] reported that these conditions represented the two most relevant factors associated with death from VL. Hemorrhagic phenomena are probably associated with disseminated intravascular coagulation [35], i.e. the activation of coagulation and fibrinolysis that is initiated as part of the inflammatory response by a mechanism similar to that established for sepsis [36].

Following a study of Tunisian children affected from VL, it was reported that bleeding and a period of more than 56 days between the onset symptoms and the first clinical examination were among the seven most important factors associated with a negative prognosis [30]. In the present study, however, the time between the onset of symptoms and clinical suspicion of VL showed no such association.

According to univariate analysis, *Leishmania*-HIV and *Leishmania*-tuberculosis co-infections were both significantly (*p*<0.05) associated with death from VL. However, in the multivariate analysis only co-infection with HIV was significantly correlated with death (ORadjusted 2.4; 95% CI 1.2–4.8). *Leishmania*-HIV co-infection is an emerging problem that requires urgent attention since, in recent years, VL has become an opportunistic disease in HIV-infected subjects. Indeed, VL may recur many times in HIV-infected patients regardless of the provision of adequate treatment, and the outcome is often fatal [37]–[40]. Moreover, the presence of VL accelerates the progression of HIV by promoting viral replication and aggravating the status of immunosuppression [40]. Additionally, it has been observed *in vitro* that HIV induces the replication of *Leishmania* by reducing T cells that are able to recognize *Leishmania* antigens. According to Cruz et al. [41], HIV may invade and replicate within *Leishmania*-bearing macrophages even though TCD4^+^ are the preferred cells. Thus, both pathogens can act in synergism and aggravate the condition of a co-infected patient [42].

In a study conducted in north-eastern Brazil involving a chronological serie of hospital records during a period of 10 years (1996–2005) and relating to 396 VL-patients (76 deaths and 320 cured patients), nine *Leishmania*-HIV co-infected individuals were identified and six of these progressed to death [25]. The authors suggested that increased lethality with co-infection could be associated with an increased risk of the disease itself or with the occurrence of other opportunistic infections. The available evidence clearly indicates that VL patients should be tested for HIV, and co-infected individuals should receive special care [12], [19], [37].

Regarding the initial drug used in treatment (Table 5), it can be observed that the fatality rate was 6.9% (42/612) for those patients treated with Pentavalent Antimonial. Among those who received Amphotericin, the case fatality rate was 28.9% (39/135). This difference demonstrated by univariate logistic regression analysis can be explained by the fact that in Brazil, the pentavalent antimonials are the drugs of choice for treatment of VL due to its proven therapeutic efficacy. Amphotericin is recommend as the first choice in patients six months or under and for those over 65 years old and those with severe clinical manifestation such as: malnutrition, comorbidities (which include bacterial infection), jaundice, hemorrhagic phenomena (except epistaxis), generalized edema, signs of toxemia (lethargy, poor perfusion, cyanosis, tachycardia or bradycardia, hypoventilation or hyperventilation, and hemodynamic instability) [19].

Measures that have been applied to control the canine reservoir and the insect vector have not been successful in preventing the spread of VL in Brazil, and early diagnose and treatment of human cases remain the main approaches for reducing lethality. Guidelines published by the Brazilian Ministry of Health stress the key factors associated with death from VL with the aim of ensuring that patients who require special care can be identified and classified according to severity of risk [12], [19]. While the results obtained in the present study support these definitions, in order to reduce death from VL it is necessary first to identify the prognosis factors and then to adopt the correct clinical strategies. As stated by Werneck et al. [4], the key challenge lies in making the correct medical decisions following the identification of high-risk patients.

The prognostic score generated in the present study is based on four clinical variables (age≥60 years, bleeding, other associated infections and jaundice). The predictive performance of this score was: sensitivity 71.4%, specificity 73.7%, positive and negative predictive value (28.9% and 94.5%) and area under the ROC curve (75.6%). The predictive performance could be improved with addition of laboratory variables according to other studies carried out in Brazil [4], [26]. However, these laboratory variables are unavailable in SINAN. The advantage of the system developed herein is its simplicity because it was based on patient age and clinical symptoms at the clinical suspicious. Additionally, decisions regarding the clinical management of VL patients can be facilitated by the identification of either a distinct prognosis factor or a combination of factors. However, further studies that advance our understanding of the factors associated with moderate and severe forms of VL are also necessary. Furthermore, it is particularly important to validate the applicability of the score in immunosuppressed VL individuals.

The key issue, however, is to define a simple prognosis score that could be applied in basic health units and would allow the early detection of VL cases for redirection to specialized health service. For the purposes of comparison, patients attaining a predictive score in the range from 1 to 5 received an allocated score of 1, while those presenting none of the death prognosis factors received an allocated score of 0. The scoring system presented here can be used to identify patients running a higher risk of death from VL at the time of clinical suspicion. Those patients with a score between 1 and 5 should receive specialized clinical management during treatment.

Our study had limitations that deserve to be discussed. The multivariate analyses did not take into account all possible factors that could contribute to unfavorable evolution of the VL case, *e.g.* nutritional status, the presence of other comorbidities (not included here as autoimmune diseases, alcoholism, and other drug abuse). These variables are not collected through the SINAN system. It is noteworthy that this study was designed to assess whether the variables in the SINAN could be useful in identifying patients facing a higher risk of death from VL at moment of clinical suspicion. However, we consider of great importance further studies designed to include, in addition to the SINAN database, clinical and laboratory variables of VL patients.

Another limitation of this study is related to the validation of the prognostic scoring system which was proposed using the same patients whose data was used in its preparation (patients in the period 2007–2009). In fact, as this score included the variables of the newer version of SINAN, there is until now no distinct set of patients for validation. In the future, we intend to validate this prognostic scoring system using several random samples from the SINAN database.

In order to improve the quality of assistance, the Brazilian Ministry of Health [12], [17], [19] has urged local surveillance authorities to provide infrastructure to the basic health units and to encourage the training of the professionals to organize comprehensive support to the patients with efficiency. Moreover, the flow of VL patients through specialized reference service and other health units must be established and disclosed. Some researchers have further suggested that VL-lethality could be greatly reduced by speeding up laboratory diagnosis and developing less toxic alternative drugs [24].

In Brazil, suspected VL cases must be notified to the health authorities using the appropriate SINAN epidemiological form. All fields in the form must be completed even in the absence of information (missing code). However, some of the variables (schooling, occupation, ethnicity, weight, date of the start of treatment and relapse) could not be evaluated in the present study because the corresponding fields had high proportion of missing data. It would have been helpful to know the date of the start of treatment, since early diagnosis and treatment of VL are important in reducing the lethality of the disease. Indeed, the absence of vital information in partially completed medical records is a chronic problem in Brazil [43].

SINAN has a specific module for VL which includes the registration of the variables described in the literature associated with the disease. In Brazil, the Epidemiological Surveillance Service works in conjunction with other information systems such as Mortality Information System (SIM). So it is routine for this service to verify if deaths from reportable diseases such as VL are listed in SINAN system. Of course, there may be some underreporting due to the difficulty in defining the VL clinical case or identifying the VL as cause of death. Therefore, underreporting is minimized by comparing the two systems mentioned.

This study may provide ammunition to the debate on the usefulness and limitations of the Reportable Disease Information System (SINAN) database. According to the World Health Organization [2], improvement in the flow of information is one of the main challenges in the control of tropical neglected diseases.

This study has identified vulnerable patients who are at higher risk of death from VL and who would benefit from early predictive evaluation of the prognostic. In conclusion the knowledge regarding the factors associated with death may contribute for clinical management and reduction of lethality from VL.

## Supporting Information

Checklist S1 **STROBE checklist.**
(DOC)
